# Correction: Detection of SARS-CoV-2 RNA by direct RT-qPCR on nasopharyngeal specimens without extraction of viral RNA

**DOI:** 10.1371/journal.pone.0240343

**Published:** 2020-10-02

**Authors:** Mohammad Rubayet Hasan, Faheem Mirza, Hamad Al-Hail, Sathyavathi Sundararaju, Thabisile Xaba, Muhammad Iqbal, Hashim Alhussain, Hadi Mohamad Yassine, Andres Perez-Lopez, Patrick Tang

The Funding statement is incorrect. The correct Funding statement is as follows: Open Access funding provided by the Qatar National Library.
