# Supplementary material for: Detection of SARS-CoV-2 RNA by direct RT-qPCR on nasopharyngeal specimens without extraction of viral RNA
Source: PLoS One. 2020 Jul 24;15(7):e0236564. doi: 10.1371/journal.pone.0236564 (PMC7380591; doi:10.1371/journal.pone.0236564)
Supplement: S1 Table — (DOCX) [file pone.0236564.s001.docx]

**S1 Table. Primers and probes used in this study**

| **Assay** | **Target** | **Name** | **Sequence (5′- 3′)** | **5’-Label** | **3’-Label** | **Final concentration (μM)** | **Source** |
| --- | --- | --- | --- | --- | --- | --- | --- |
| HCoV-HKU1 | Nucleocapsid gene | HCoV-HKU1-F | GATCCTACTAYTCAAGAAGCTATCCCTACT |  |  | 0.6 | (12) |
|  |  | HCoV-HKU1-R | ACCTTCCTGAGCCTTCAACATAAT |  |  | 0.3 |  |
|  |  | HCoV-HKU1-P | TTTYCGCCTGGTACGATTTTGCCTC | JOE/ZEN | IaBkFQ | 0.2 |  |
| SARS-CoV-2 | E gene | E_Sarbeco_F1 | ACAGGTACGTTAATAGTTAATAGCGT |  |  | 0.4 | (4) |
|  |  | E_Sarbeco_R2 | ATATTGCAGCAGTACGCACACA |  |  | 0.4 |  |
|  |  | E_Sarbeco_P1 | ACACTAGCCATCCTTACTGCGCTTCG | FAM/ZEN | IaBkFQ | 0.2 |  |
|  | Internal control | MS2-TM3-F | GGCTGCTCGCGGATACCC |  |  | 0.2 | (14) |
|  |  | MS2-TM3-R | TGAGGGAATGTGGGAACCG |  |  | 0.2 |  |
|  |  | MS2-TM2JOE | ACCTCGGGTTTCCGTCTTGCTCGT | JOE/ZEN | IaBkFQ | 0.1 |  |
|  | RNaseP | RNaseP-F | CCAAGTGTGAGGGCTGAAAAG |  |  | 0.3 | (13) |
|  |  | RNaseP-R | TGTTGTGGCTGATGAACTATAAAAGG |  |  | 0.3 |  |
|  |  | RNaseP-P | CCCCAGTCTCTGTCAGCACTCCCTTC | JOE/ZEN | IaBkFQ | 0.2 |  |
