# Supplementary material for: Detection of SARS-CoV-2 RNA by direct RT-qPCR on nasopharyngeal specimens without extraction of viral RNA
Source: PLoS One. 2020 Jul 24;15(7):e0236564. doi: 10.1371/journal.pone.0236564 (PMC7380591; doi:10.1371/journal.pone.0236564)
Supplement: S2 Table — NPFS specimens were either i) subjected to viral RNA extraction by standard method using a NucliSENS easyMAG automated extraction system (Biomerieux), or ii) serially diluted with nuclease free water (NFW) followed by incubation at 100°C for 5 minutes, or iii) freeze thawed once prior to heat treatment. All heat-treated samples were centrifuged at 13,000 rpm for 5 minutes at 4°C and supernatants were collected. All samples were tested for SARS-CoV-2 RNA by standard RT-qPCR in duplicate and mean CT values were compared. (DOCX) [file pone.0236564.s002.docx]

**S2 Table. Direct RT-qPCR on a nasopharyngeal specimen positive for human coronavirus HKU1 after heat treatment at 100^o^C for 5 minutes**

| **Sample Name** | **Sample dilution** | **Sample processing** | **HKU1 CoV C_T_** | |
| --- | --- | --- | --- | --- |
|  |  |  | **Quantifast Pathogen RT-PCR + IC Master Mix** | **TaqPath™ 1-Step RT-qPCR Master Mix** |
| 1167-H0 | Undiluted | easyMAG extraction | 24.6 | 23.8 |
| 1167-H1 | Undiluted | Heat treatment | 36.4 | 36.6 |
| 1167-H2 | 2 fold dilution | Heat treatment | 34.5 | 35.7 |
| 1167-H3 | 4 fold dilution | Heat treatment | 34.7 | 35.6 |
| 1167-H4 | 10 fold dilution | Heat treatment | 34.1 | 33.2 |
| 1167-H5 | Undiluted | Freeze thaw; heat treatment | 38.1 | 36.1 |

NPFS specimens were either i) subjected to viral RNA extraction by standard method using a NucliSENS easyMAG automated extraction system (Biomerieux), or ii) serially diluted with nuclease free water (NFW) followed by incubation at 100^o^C for 5 minutes, or iii) freeze thawed once prior to heat treatment. All heat-treated samples were centrifuged at 13,000 rpm for 5 minutes at 4^o^C and supernatants were collected. All samples were tested for SARS-CoV-2 RNA by standard RT-qPCR in duplicate and mean C_T_ values were compared.
