# Supplementary material for: Detection of SARS-CoV-2 RNA by direct RT-qPCR on nasopharyngeal specimens without extraction of viral RNA
Source: PLoS One. 2020 Jul 24;15(7):e0236564. doi: 10.1371/journal.pone.0236564 (PMC7380591; doi:10.1371/journal.pone.0236564)
Supplement: S3 Table — Specimens were collected from laboratory members who volunteered to provide specimens. NPFS specimens were either i) collected in VTM or ii) collected in sterile empty tubes as dry swabs and later resuspended in 1 ml of NFW. Five μl of a patient specimen positive for SARS-CoV-2 RNA (CT = 22) were spiked into 0.2 ml of specimens collected in VTM of NFW. VTM specimens were extracted by standard method using a NucliSENS easyMag automated extraction system (bioMerieux). Specimens collected in NFW were incubated at 100°C for 5 minutes and centrifuged at 13,000 rpm for 5 minutes at 4°C and supernatants were collected. All samples were tested for SARS-CoV-2 RNA by standard RT-qPCR using Quantifast Pathogen RT-PCR + IC Master Mix in duplicate and mean CT values were compared. (DOCX) [file pone.0236564.s003.docx]

**S3 Table. Direct RT-qPCR on simulated, dry NPFS using a spiked specimen positive for SARS-CoV-2**

| **Sample No.** | **SARS-CoV-2 C_T_** | |
| --- | --- | --- |
|  | **Standard method** | **Direct PCR on dry NPFS** |
| 1 | 20.8 | 32.4 |
| 2 | 20.5 | 25.8 |
| 3 | 20.8 | 32.4 |
| 4 | 25.7 | 31.6 |
| 5 | 25.8 | 33.8 |
| 6 | 25.6 | 34.5 |

Specimens were collected from laboratory members who volunteered to provide specimens. NPFS specimens were either i) collected in VTM or ii) collected in sterile empty tubes as dry swabs and later resuspended in 1 ml of NFW. Five μl of a patient specimen positive for SARS-CoV-2 RNA (C_T_ = 22) were spiked into 0.2 ml of specimens collected in VTM of NFW. VTM specimens were extracted by standard method using a NucliSENS easyMag automated extraction system (bioMerieux). Specimens collected in NFW were incubated at 100^o^C for 5 minutes and centrifuged at 13,000 rpm for 5 minutes at 4^o^C and supernatants were collected. All samples were tested for SARS-CoV-2 RNA by standard RT-qPCR using Quantifast Pathogen RT-PCR + IC Master Mix in duplicate and mean C_T_ values were compared.
