# Supplementary material for: Detection of SARS-CoV-2 RNA by direct RT-qPCR on nasopharyngeal specimens without extraction of viral RNA
Source: PLoS One. 2020 Jul 24;15(7):e0236564. doi: 10.1371/journal.pone.0236564 (PMC7380591; doi:10.1371/journal.pone.0236564)
Supplement: S5 Table — NPFS specimens were processed according to manufacturer’s protocol (PrimeDirect™ Probe RT-qPCR Mix, Takara Bio Inc.). All samples were tested for SARS-CoV-2 RNA by standard RT-qPCR in duplicate and mean CT values were compared. (DOCX) [file pone.0236564.s005.docx]

**S5 Table. Direct RT-qPCR on SARS-CoV-2 positive and negative NPFS specimens processed using Takara PrimeDirect Probe RT-qPCR Mix**

| **Sample No.** | **SARS-CoV-2 C_T_** | |
| --- | --- | --- |
|  | **Standard method** | **Takara PrimeDirect protocol** |
| 1 | 21.5 | 29.78692245 |
| 2 | 34.5 | Undetermined |
| 3 | 24.5 | Undetermined |
| 4 | 33.7 | Undetermined |
| 5 | Undetermined | Undetermined |

NPFS specimens were processed according to manufacturer’s protocol (PrimeDirect™ Probe RT-qPCR Mix, Takara Bio Inc.). All samples were tested for SARS-CoV-2 RNA by standard RT-qPCR in duplicate and mean C_T_ values were compared.
