# Supplementary material for: Detection of SARS-CoV-2 RNA by direct RT-qPCR on nasopharyngeal specimens without extraction of viral RNA
Source: PLoS One. 2020 Jul 24;15(7):e0236564. doi: 10.1371/journal.pone.0236564 (PMC7380591; doi:10.1371/journal.pone.0236564)
Supplement: S6 Table — NPFS specimens were either i) subjected to viral RNA extraction by standard method using a NucliSENS easyMAG automated extraction system (bioMerieux), or ii) diluted 4-fold with nuclease free water (NFW) iii) diluted 4-fold with nuclease free water (NFW) followed by incubation at 65°C for 5 minutes or iv) diluted 4-fold with Tween-20 to final concentration of 0.2% followed by incubation at room temperature for 10 min. All samples were tested for SARS-CoV-2 RNA by standard RT-qPCR using Quantifast Pathogen RT-PCR + IC Master Mix. (DOCX) [file pone.0236564.s006.docx]

**S6 Table. Direct RT-qPCR on SARS-CoV-2 positive and negative NPFS specimens with or without heating at 65^o^C for 5 minutes or after lysis using a non-ionic detergent**

| **Sample No.** | **SARS-CoV-2 C_T_** | | | |
| --- | --- | --- | --- | --- |
|  | **Standard method** | **No treatment** | **Heat treatment** | **Lysis with 0.2% Tween-20** |
| 1 | 21.5 | 30.6 | 26.7 | 28.4 |
| 2 | 34.5 | Undetermined | Undetermined | Undetermined |
| 3 | Undetermined | Undetermined | Undetermined | Undetermined |
| 4 | Undetermined | Undetermined | Undetermined | Undetermined |
| 5 | Undetermined | Undetermined | Undetermined | Undetermined |

NPFS specimens were either i) subjected to viral RNA extraction by standard method using a NucliSENS easyMAG automated extraction system (bioMerieux), or ii) diluted 4-fold with nuclease free water (NFW) iii) diluted 4-fold with nuclease free water (NFW) followed by incubation at 65^o^C for 5 minutes or iv) diluted 4-fold with Tween-20 to final concentration of 0.2% followed by incubation at room temperature for 10 min. All samples were tested for SARS-CoV-2 RNA by standard RT-qPCR using Quantifast Pathogen RT-PCR + IC Master Mix.
