# Supplementary material for: Detection of SARS-CoV-2 RNA by direct RT-qPCR on nasopharyngeal specimens without extraction of viral RNA
Source: PLoS One. 2020 Jul 24;15(7):e0236564. doi: 10.1371/journal.pone.0236564 (PMC7380591; doi:10.1371/journal.pone.0236564)
Supplement: S7 Table — QCMD EQA specimens were simultaneously tested by standard and direct approach and by QIAstat-Dx Respiratory 2019-nCoV Panel (Qiagen). (DOCX) [file pone.0236564.s007.docx]

**S7 Table. Direct RT-qPCR on QCMD EQA specimens**

| **Sample No.** | **QCMD result** | **SARS-CoV-2 C_T_** | | |
| --- | --- | --- | --- | --- |
|  |  | **Standard approach** | **QIAstat** | **Direct approach** |
| Sample 1 | Positive | 33.4 | 34 | 35.5 |
| Sample 2 | Negative | undetermined | undetermined | Undetermined |
| Sample 3 | Positive | 35.2 | 35.4 | 37.1 |
| Sample 4 | Negative | undetermined | undetermined | Undetermined |
| Sample 5 | Negative | undetermined | undetermined | Undetermined |
| Sample 6 | Positive | 34.2 | 36.7 | 35.1 |
| Sample 7 | Positive | 29.4 | 31.5 | 31.7 |
| Sample 8 | Borderline | undetermined | undetermined | Undetermined |

QCMD EQA specimens were simultaneously tested by standard and direct approach and by QIAstat-Dx Respiratory 2019-nCoV Panel (Qiagen)
