# Supplementary material for: Detection of SARS-CoV-2 RNA by direct RT-qPCR on nasopharyngeal specimens without extraction of viral RNA
Source: PLoS One. 2020 Jul 24;15(7):e0236564. doi: 10.1371/journal.pone.0236564 (PMC7380591; doi:10.1371/journal.pone.0236564)
Supplement: S8 Table — (DOCX) [file pone.0236564.s008.docx]

**S8 Table. Detection of SARS-CoV-2 with RNaseP as endogenous control by direct RT-qPCR and standard methods in NPFS specimens**

| **Sample No.** | **SARS-CoV-2** | | | **RNaseP** | | |
| --- | --- | --- | --- | --- | --- | --- |
|  | **Extracted** | **Direct** | **ΔC_T_** | **Extracted** | **Direct** | **ΔC_T_** |
| 1 | Undetermined | Undetermined | - | 28.3 | 32.7 | 4.4 |
| 2 | Undetermined | Undetermined | - | 25.4 | 29.6 | 4.2 |
| 3 | Undetermined | Undetermined | - | 27.7 | 33.7 | 6.1 |
| 4 | Undetermined | Undetermined | - | 26.4 | 28.8 | 2.3 |
| 5 | Undetermined | Undetermined | - | 26.4 | 33.8 | 7.4 |
| 6 | Undetermined | Undetermined | - | 28.1 | 33.8 | 5.7 |
| 7 | Undetermined | Undetermined | - | 26.3 | 27.0 | 0.7 |
| 8 | Undetermined | Undetermined | - | 29.4 | 31.7 | 2.3 |
| 9 | Undetermined | Undetermined | - | 26.9 | 32.5 | 5.7 |
| 10 | Undetermined | Undetermined | - | 27.8 | 30.6 | 2.8 |
| 11 | 31.5 | 36.1 | 4.6 | 28.6 | 35.3 | 6.7 |
| 12 | 17.9 | 19.6 | 1.7 | 23.2 | 24.5 | 1.4 |
| 13 | Undetermined | Undetermined | - | 25.8 | 30.0 | 4.2 |
| 14 | Undetermined | Undetermined | - | 24.1 | 28.6 | 4.6 |
| 15 | 36.3 | Undetermined | - | 24.9 | 32.2 | 7.4 |
| 16 | Undetermined | Undetermined | - | 22.7 | 26.8 | 4.1 |
| 17 | 22.2 | 23.3 | - | 24.9 | 27.5 | 2.6 |
| 18 | Undetermined | Undetermined | - | 25.3 | 29.0 | 3.7 |
| 19 | Undetermined | Undetermined | - | 24.9 | 30.1 | 5.2 |
| 20 | 24.0 | 25.8 | 1.8 | 24.2 | 27.6 | 3.4 |
| 21 | Undetermined | Undetermined | - | 23.5 | 29.5 | 6.0 |
| 22 | Undetermined | Undetermined | - | 26.8 | 30.6 | 3.8 |
| 23 | 24.3 | 24.6 | 0.3 | 22.4 | 25.7 | 3.2 |
| 24 | Undetermined | Undetermined | - | Undetermined | 23.9 | - |
| 25 | 20.5 | 22.4 | 1.9 | 23.4 | 26.7 | 3.3 |
| 26 | Undetermined | Undetermined | - | 27.8 | 31.4 | 3.5 |
| 27 | 22.7 | 25.2 | 2.5 | 28.3 | 31.7 | 3.5 |
| 28 | Undetermined | Undetermined | - | Undetermined | 28.4 | - |
| 29 | 28.8 | 31.1 | 2.3 | 27.0 | 33.0 | 6.0 |
| 30 | Undetermined | Undetermined | - | 22.3 | 25.7 | 3.5 |
